# Mitoquinone Can Effectively Improve the Quality of Thawed Boar Sperm

**DOI:** 10.3390/ani15192808

**Published:** 2025-09-26

**Authors:** Yingying Dong, Qian Wang, Hechuan Wang, Qing Guo, Yanbing Li, Jingchun Li

**Affiliations:** College of Animal Science and Veterinary Medicine, Heilongjiang Bayi Agricultural University, Daqing 163319, China; dyy19980906@163.com (Y.D.); 19845296062@163.com (Q.W.); wanghechuan1999@outlook.com (H.W.); qguo89@126.com (Q.G.); liyanbing929@163.com (Y.L.)

**Keywords:** cryopreservation, boar sperm, MitoQ

## Abstract

**Simple Summary:**

During the freezing preservation process, boar sperm is susceptible to attack by reactive oxygen species, which can induce sperm oxidative stress and consequently lead to a decline in sperm quality. Research has found that Mitoquinone possesses significant antioxidant activity and can notably improve mitochondrial function in sperm. This study aims to evaluate the effects of adding Mitoquinone to the cryodiluent on the quality, antioxidant capacity, and mitochondrial activity of sperm after freezing and thawing. The results indicate that Mitoquinone significantly improved sperm motility and viability, and also increased acrosome reaction, membrane integrity, and mitochondrial activity. Furthermore, Mitoquinone can significantly enhance the antioxidant capacity of sperm, reducing oxidative stress-induced damage to the sperm. In this study, the optimal concentration of Mitoquinone was found to be 150 nmol/L.

**Abstract:**

Boar sperm is susceptible to damage by reactive oxygen species during in vitro preservation, leading to lipid peroxidation, which changes the sperm structure and affects its quality after thawing. Exogenous antioxidants play a vital role in preventing this damage. This research aimed to assess the impact of incorporating Mitoquinone into cryopreservation extenders on the quality and antioxidant capacity of boar sperm. Mitoquinone was added to the cryopreservation extender at varying concentrations, namely, 0, 50, 100, 150, and 200 nmol/L. Post-thawing, the sperm were examined for motility parameters, acrosome integrity, DNA integrity, mitochondrial activity, membrane integrity, and antioxidant enzyme activity. The results showed that compared with the control group, 150 nmol/L Mitoquinone could improve sperm viability after freezing and thawing and significantly reduce the malformation rate (*p* < 0.05). The addition of 150 nmol/L Mitoquinone led to a significant increase in the acrosome integrity, DNA integrity, mitochondrial activity, and membrane integrity of the boar sperm compared to the control group (*p* < 0.05). Moreover, it enhanced the antioxidant capacity of the sperm. This study demonstrated that the cryopreservation extender containing 150 nmol/L of Mitoquinone can enhance the effectiveness of semen cryopreservation.

## 1. Introduction

Artificial insemination (AI) is a reproductive biotechnology of great significance, accounting for an increasing proportion of livestock reproduction, genetic improvement, and breeding [1]. Fertility is generally lower after insemination with frozen semen compared to fresh semen. This is due to the physical damage caused by changes in osmotic pressure, the formation of ice crystals in cells, and oxidative stress caused by excessive production of ROS, which leads to the deterioration of sperm function and sperm quality after thawing [2]. Low levels of ROS regulate processes such as sperm capacitation and acrosome responses. When ROS production exceeds the body’s ability to clear it, sperm motility is reduced, leading to lipid membrane peroxidation [3]. Boar sperm plasma membranes have significantly more polyunsaturated fatty acids than sperm from other species, and their intrinsic antioxidant defense capacity is relatively limited, which will be further weakened with the dilution of sperm [4].

Mitochondria serve as the crucial sites for cellular metabolism and physiological processes. They play a crucial role in metabolism and ATP production through oxidative phosphorylation (OXPHOS) [5]. However, the accumulation of excess ROS results in an imbalance in the oxidative–antioxidant system of sperm [6], leading to reduced mitochondrial activity [7]. Under normal circumstances, reactive oxygen species (ROS), as by-products of sperm aerobic metabolism, are crucial for physiological processes of sperm, such as capacitation and interaction with oocytes. However, when the levels of ROS exceed the antioxidant capacity of sperm, oxidative stress can impair sperm physiological function through oxidative damage, potentially leading to mitochondrial dysfunction in sperm and, consequently, a decline in sperm quality [8]. Thus, enhancing the antioxidant capacity of mitochondria represents a potential approach for decreasing the production of ROS. Antioxidants targeted toward mitochondria can specifically safeguard mitochondria against damage caused by oxidative stress.

**Figure 1:**
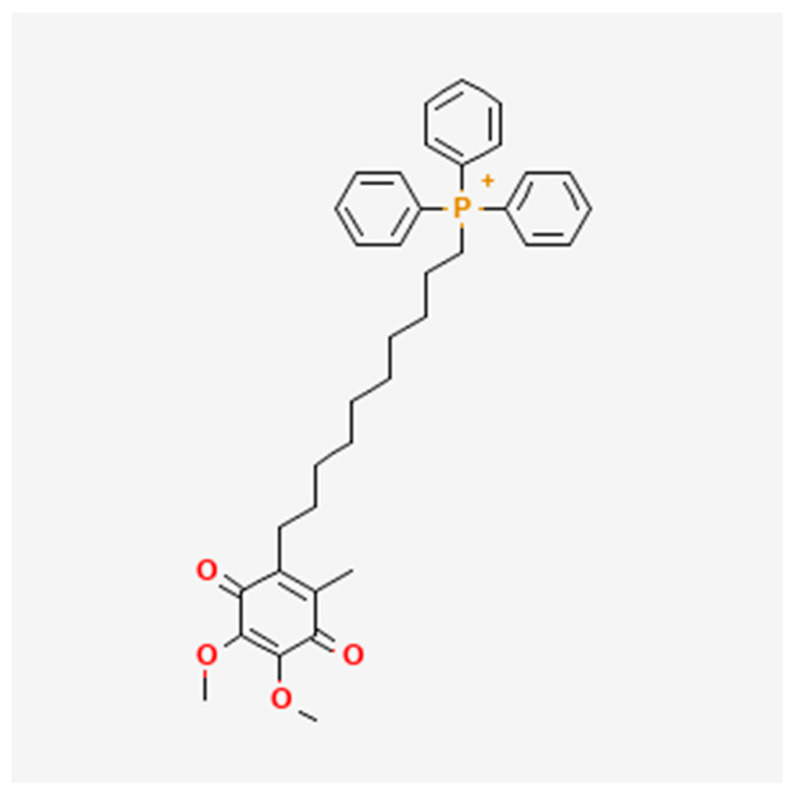
The chemical structure of MitoQ (https://pubchem.ncbi.nlm.nih.gov/compound/11388332, accessed on 9 May 2025) [9].

Mitoquinone (MitoQ) (Figure 1), composed of triphenylphosphine (TPP) and ubiquinone (In MitoQ, TPP and ubiquinone are combined in a weight ratio of 1:1) [9], acts as a selective inhibitor of mitochondrial ROS, effectively reducing the production of ROS and superoxide anions [10]. It is considered a typical mitochondrial-targeted antioxidant [11]. The addition of this antioxidant can enhance mitochondrial activity by limiting cell apoptosis, regulating cellular stress, and reducing lipid peroxidation [12]. Hatami et al. [13] discovered that MitoQ can reduce oxidative stress and improve the quality of frozen semen in male deer during the cryopreservation process by regulating mitochondrial function. Furthermore, it holds a positive charge and, while traversing the mitochondrial inner membrane respiratory chain, converts ubiquinone to ubiquinol, functioning as an antioxidant [14]. MitoQ has been validated in various animal models, demonstrating its ability to reduce oxidative stress levels by alleviating mitochondrial damage [15]. Recent studies have found that the addition of MitoQ to the semen cryopreservation diluent for ram sperm can reduce ROS production and improve sperm motility after thawing [16]. Similar studies have also demonstrated that the addition of low concentrations of MitoQ during the freezing process of stallion semen can improve sperm motility after thawing [17]. In vitro fertilization studies on cattle [18] and mice [19] have shown that the addition of MitoQ can improve oocyte maturation and developmental competence, as well as increase blastocyst yield.

According to the current literature, there are no studies on the protective effect and antioxidant capacity of MitoQ on frozen–thawed boar sperm. This investigation aimed to examine the influence that supplementing MitoQ has on the quality and antioxidant properties of boar semen during cryopreservation.

## 2. Materials and Methods

### 2.1. Ethics Approval

This study has received approval from the Animal Experiments Committee of Heilongjiang Bayi Agricultural University (License No. DWKJXY2023058). 

### 2.2. Preparation of Reagents and Extenders

MitoQ used in this experiment was purchased from Shanghai MCE (Med Chem Express, Shanghai, China). Unless stated otherwise, all chemicals were obtained from Sigma-Aldrich (St. Louis, MO, USA).

The thawing agent and cryopreservation extenders (1) and (2) were prepared according to the methods of Zhang et al. [20]. Cryopreservation extender (1) consisted of 197.64 mmol/L lactose, 1 million U/L streptomycin, 106.92 mmol/L gentamicin, and 200 mL/L egg yolk. Cryopreservation extender (2) was prepared by adding 3% glycerol to extender (1) and adjusting the pH to 7.2–7.4. The prepared extenders were centrifuged using a Sigma Laborzentrifuge (Sankt Augustin, Germany) at 4 °C for 10 min at 16,770× *g* to remove egg yolk residue, and were then stored at 4 °C.

Concentrations of 0, 50, 100, 150, and 200 nmol/L MitoQ were mixed with cryopreservation extenders (1) or (2). The thawing agent for the cryopreserved sperm contained 124.61 mmol/L fructose, ethylenediamine 95.18 mmol/L tris(hydroxymethyl)aminomethane, 3.08 mmol/L sodium bicarbonate, 26.37 mmol/L caffeine, and 3.82 mmol/L sodium pyruvate, with a pH of 7.3 ± 0.1.

### 2.3. Semen Collection and Processing

#### 2.3.1. Sperm Collection

Ten 3-year-old Duroc boars were selected from a commercial boar station (Jingyu Animal Husbandry Station in Daqing, China) for semen collection. Semen collection was performed once a week using the gloved hand method for four consecutive weeks, with the mid-ejaculate collected each time. The collected semen was mixed thoroughly, placed in a 37 °C incubator, and transported to the laboratory. The initial quality inspection of the sperm was performed using a Computer-Assisted Sperm Analysis system CASA3.0 (Nanjing Songjing Tianlun, Nanjing, China). Semen samples containing sperm with a viability of >85%, and with no abnormal odor and normal color, were selected for subsequent experiments.

#### 2.3.2. Sperm Cryopreservation and Thawing Process

Semen samples were centrifuged at 290× *g* (Cence Laboratory Instruments, Changsha, China) for 10 min, and the supernatants were discarded. The sperm were then resuspended in cryopreservation extender (1) to a concentration of 4 × 10^8^/mL and equilibrated at 17 °C for 20 min. Subsequently, the sperm concentration was adjusted to 2 × 10^8^/mL using cryopreservation extender (2), and the sperm samples were placed in a 4 °C incubator for 60 min. The extended sperm samples were then loaded into 0.5 mL sperm straws. On each sampling day, a total of 100 sperm straws were filled, with a mean of 20 sperm straws per group. The straws were then placed in the cryogenic programming instrument.

Sperm freezing and thawing were performed according to the methods of Baishya et al. [21], with appropriate modifications. The freezing regimen was 5 °C to −10 °C with cooling at a rate of −5 °C/min; −10 °C to −100 °C at −40 °C/min; and −100 °C to −140 °C at −20 °C/min. Immediately after freezing, the straws were placed in a liquid nitrogen tank (−196 °C) and stored for 30 days. To thaw the sperm, the straws were rapidly removed from the liquid nitrogen and placed in a 50 °C water bath for 16 s. The sperm were then thoroughly mixed with 400 µL of thawing agent, and the mixture was placed in a 37 °C incubator, ahead of testing.

### 2.4. Sperm Quality Test

#### 2.4.1. Detection of Sperm Quality and Kinetic Parameters

Sperm quality and kinetic parameters were assessed using the Mailang automatic sperm analysis system CASA (ML-608JZ; Songjing Tianlun Biotechnology, Nanning, China). The sperm concentration was adjusted to 1 × 10^8^/mL using the thawing solution, and 10 µL samples were placed on glass slides prewarmed to 37 °C and covered with coverslips. Five fields of view were randomly selected under a 200× magnification microscope (ML-800 II; Songjing Tianlun Biotechnology, Nanning, China), with each field containing ≥150 sperm. The kinetic parameters assessed included curvilinear velocity (VCL [µm/s]), average path velocity (VAP [µm/s]), straight-line velocity (VSL [µm/s]), and beat cross frequency (BCF [Hz]). The CASA system settings were as follows: minimum frame rate: 30 Hz; VCL activity limit: 60 µm/s; VSL activity limit: 60 µm/s; VAP activity limit: 50 µm; BCF activity limit: 100 Hz; maximum movement speed: 100 µm/s; and minimum movement speed: 30 µm/s.

#### 2.4.2. Detection of Sperm Acrosome Integrity and Plasma Membrane Integrity

According to the research report by Spinaci et al. [22], the integrity of the sperm acrosome was detected using the fluorescein isothiocyanate-conjugated peanut agglutinin (FITC-PNA) staining method. In summary, 20 µL of the sperm sample was spread onto a clean glass slide and allowed to air-dry. The samples were then fixed with methanol for 10 min and allowed to air-dry again. Next, 20 µL of the FITC-PNA working solution was evenly dispensed onto the air-dried sperm samples, and the slides were incubated in the dark at 37 °C for 30 min. After incubation, the samples were rinsed with PBS and allowed to air-dry in the dark. A 200× fluorescence microscope (EVOS FL, Thermo Fisher Scientific, Waltham, MA, USA) was used to observe at least five fields of view, ensuring that the total number of sperm was no fewer than 300.

According to the report by Gliozzi et al. [23], the integrity of the sperm membrane was assessed using a dual staining method with SYBR-14 and propidium iodide (PI). Briefly, 200 µL of sperm sample was centrifuged at 290× *g* for 5 min, and the supernatant was discarded. Then, 150 µL of PBS was added to the sedimented sperm sample, followed by 5 µL of SYBR-14 and 5 µL of PI working solution, with thorough mixing. The mixture was incubated in the dark at 37 °C for 5 min. A 10 µL sample was then taken, dropped onto a glass slide, and observed under a 200× fluorescence microscope. At least five fields of view were observed, and no fewer than 300 sperm were counted.

#### 2.4.3. Detection of Sperm Mitochondrial Activity and DNA Integrity

We used the JC-I staining method to evaluate the mitochondrial activity of the sperm samples [24]. In brief, 250 µL of sperm sample was centrifuged at 290× *g* for 5 min and the supernatant was discarded. Next, 125 µL of PBS was added to the pellet, followed by 5 µL of the JC-1 working solution. The resulting mixture was then incubated in the dark at 37 °C for 30 min. Next, we took a 10 µL sample and dropped it onto a glass slide, then observed it using a 200× fluorescence microscope. At least five fields of view were observed, and the total number of sperm was no less than 300.

Sperm DNA integrity was assessed using the acridine orange (AO) staining method [25]. Briefly, 30 µL of the sperm sample was spread onto a clean glass slide and allowed to air-dry. The samples were then fixed in methanol for 10 min, followed by air-drying. Next, 20 µL of AO working solution was added to the air-dried sperm samples, and the slides were incubated in the dark at 37 °C for 30 min. Post-incubation, the samples were rinsed with HEPES buffer and air-dried in the dark. Finally, sperm DNA integrity was evaluated under a fluorescence microscope at 200× magnification, with at least five fields of view examined and a minimum of 300 sperm counted per sample.

### 2.5. Detection of Sperm Antioxidant Capacity

The antioxidant capacity of sperm was detected using an antioxidant kit [26]. Commercial biochemical kits from the Jiancheng Bioengineering Institute in Nanjing, China, were employed to determine multiple antioxidant parameters of sperm samples. These parameters included the activity of superoxide dismutase (SOD) and catalase (CAT); the content of malondialdehyde (MDA), glutathione peroxidase (GSH-Px), and hydrogen peroxide (H_2_O_2_); and the total antioxidant capacity (T-AOC). All sample preparations strictly followed the operating instructions of the kits. A spectrophotometer (INESA, Shanghai, China) was used to measure the samples. The absorbance was measured at 522 nm for MDA, at 593 nm for T-AOC, and at 405 nm for GSH-Px, H_2_O_2_, and CAT. Meanwhile, a microplate reader (BIO-RAD, Hercules, CA, USA) was employed to measure SOD activity at 450 nm.

### 2.6. Detection of Apoptotic Proteins in Thawed Sperm by Western Blotting

The levels of Bcl-xl, BAK, and Bcl-2 in thawed sperm were detected by Western blotting. Briefly, semen samples were lysed with RIPA protein lysis buffer (Catalog No. P0013B, Beyotime Biotechnology, Shanghai, China). Fifty micrograms of protein were taken and separated by 10% sodium dodecyl sulfate-polyacrylamide gel electrophoresis (SDS-PAGE). Then, the proteins were transferred to a nitrocellulose membrane (Solarbio, Beijing, China). Rabbit-derived anti-Bcl-xl antibody (ab32370, Abcam, Cambridge, UK), rabbit-derived anti-BAK antibody (ab32423, Abcam), and rabbit-derived anti-Bcl-2 antibody (ab32124, Abcam) were all diluted at a ratio of 1:1500. Before visual detection of reactive proteins using chemiluminescent (ECL) protein immunoblotting reagents and the ChemiDoc XRS imaging system (Image Lab 6.0) (Bio-Rad Laboratories, Inc., Hercules, CA, USA), goat anti-rabbit antibodies labeled with horseradish peroxidase (HRP) were incubated at a ratio of 1:12,000 for 1.5 h.

### 2.7. Sperm Penetration Experiment

According to the method described by Yanagimachi et al. [27], the ability of sperm to penetrate the oocyte membrane was assessed. Forty IU of follicle-stimulating hormone (PMSG) was injected into the abdominal cavity of 8–12-week-old female golden hamsters on the first day of their estrous cycle. After 54–58 h, 30 IU of human chorionic gonadotropin (hCG) was injected, followed by oocyte collection after a 17 h interval. The collected oocytes were divided into groups of 10–15, and each group was co-incubated with 1 × 10^6^ thawed sperm. The culture surface was sealed with mineral oil, and after 6 h of incubation in a 37 °C, 5% CO_2_ incubator, sperm penetration into the oocytes was carefully observed. To facilitate observation, sperm outside the transparent zone were digested using 0.25% hyaluronidase.

### 2.8. Statistical Analysis

IBM SPSS 26 software was employed to conduct one-way ANOVA and Duncan’s multiple tests for data analysis. All data from each experiment were tested for normality with the one-sample Kolmogorov–Smirnov test. If some data did not show normality, we arcsine-transformed the variable before the analysis and again checked the normality using the one-sample Kolmogorov–Smirnov test for this parameter. Data analysis tests were conducted multiple times. The outcomes are presented in the form of mean ± standard deviation. Statistical significance was determined when *p* < 0.05.

## 3. Results

### 3.1. The Influence of MitoQ on the Post-Thawing Kinetic Parameters

Consistent with the findings of other studies, the cryopreservation of semen will lead to a decrease in sperm motility and a reduction in semen quality after thawing. Adding MitoQ to the cryopreservation extender can significantly increase the post-thaw viability of sperm and kinetic parameters. Adding 150 nmol/L MitoQ significantly improved sperm motility and viability compared to 0 nmol/L MitoQ (*p* < 0.05). The kinetic parameters of the boar sperm treated with MitoQ were significantly higher than those in the 0 nmol/L MitoQ group (*p* < 0.05). Among them, the sperm treated with 150 nmol/L MitoQ showed the best effects in terms of the VAP, VSL, and VCL (*p* < 0.05). Compared to the 0 nmol/L MitoQ group, the sperm BCF of the 150 nmol/L MitoQ group significantly increased (*p* < 0.05), and the rate of sperm malformation significantly decreased (*p* < 0.05) (Table 1).

### 3.2. The Influence of MitoQ on the Post-Thawing Acrosome Integrity and Membrane Integrity

**Figure 2:**
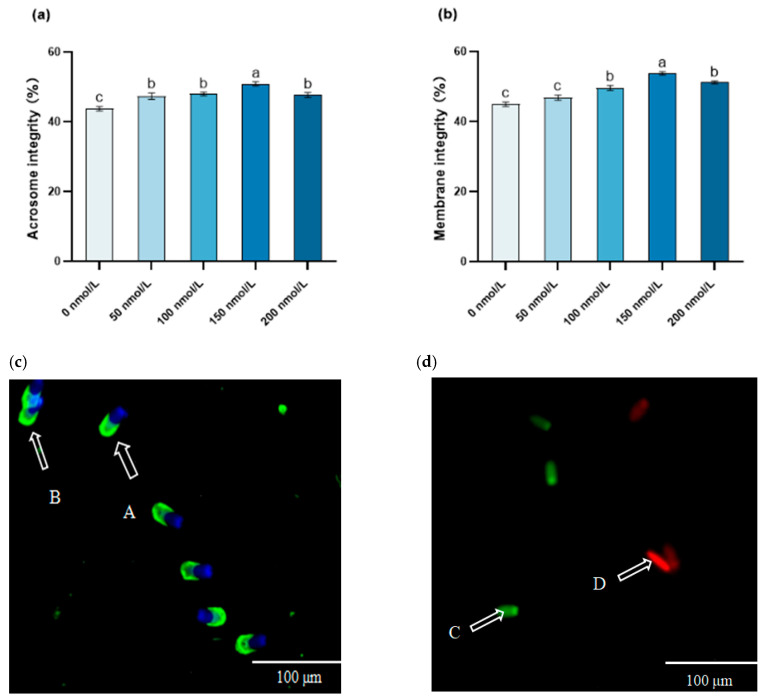
Effect of MitoQ on acrosome integrity and membrane integrity of boar sperm. (**a**) Acrosome integrity; (**b**) membrane integrity; (**c**) images of sperm acrosome staining: A: sperm with a complete acrosome, B: damaged sperm with a compromised acrosome; (**d**) images of sperm plasma membrane staining: C: integrity of the membrane, D: cytoplasmic membrane rupture. The different lowercase letters indicate *p* <  0.05.

Compared with the treatment groups receiving MitoQ at other concentrations, the sperm acrosome integrity in the 150 nmol/L MitoQ group was significantly higher than that in the 0 nmol/L MitoQ group (*p* < 0.05). In contrast to the 50 nmol/L and 100 nmol/L MitoQ groups, the acrosome integrity of the sperm in the 200 nmol/L MitoQ group was significantly higher (*p* < 0.05). Moreover, in comparison to the 0 nmol/L MitoQ group, the membrane integrity of the sperm in the 150 nmol/L MitoQ group was notably elevated (*p* < 0.05) (Figure 2).

### 3.3. The Influence of MitoQ on the Post-Thawing Mitochondrial Activity and DNA Integrity

**Figure 3:**
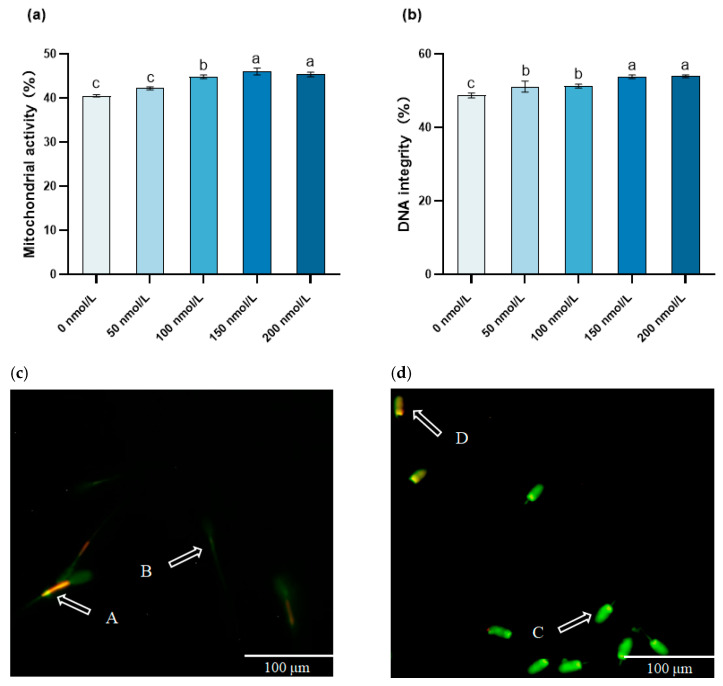
Effect of MitoQ on mitochondrial activity and DNA integrity in boar sperm. (**a**) Mitochondrial activity; (**b**) DNA integrity; (**c**) mitochondrial staining images: A: highly active mitochondria, B: low-activity mitochondria; (**d**) image of DNA integrity: C: DNA-intact spermatozoa, D: sperm with DNA damage. The different lowercase letters indicate *p* < 0.05.

The mitochondrial activity of the 150 and 200 nmol/L MitoQ groups was significantly higher than that of the other MitoQ-treated groups at other concentrations (*p* < 0.05). Specifically, compared with the 0 nmol/L MitoQ group, the mitochondrial activity of the sperm in the 150 nmol/L MitoQ group was significantly higher (*p* < 0.05). The sperm treated with 150 and 200 nmol/L MitoQ demonstrated significantly greater DNA integrity (*p* < 0.05). Additionally, the mitochondrial activity in the 50 and 100 nmol/L MitoQ groups was significantly higher than that in the 0 nmol/L MitoQ group (*p* < 0.05) (Figure 3).

### 3.4. The Influence of MitoQ on the Post-Thawing Antioxidant Enzyme Activity

**Figure 4:**
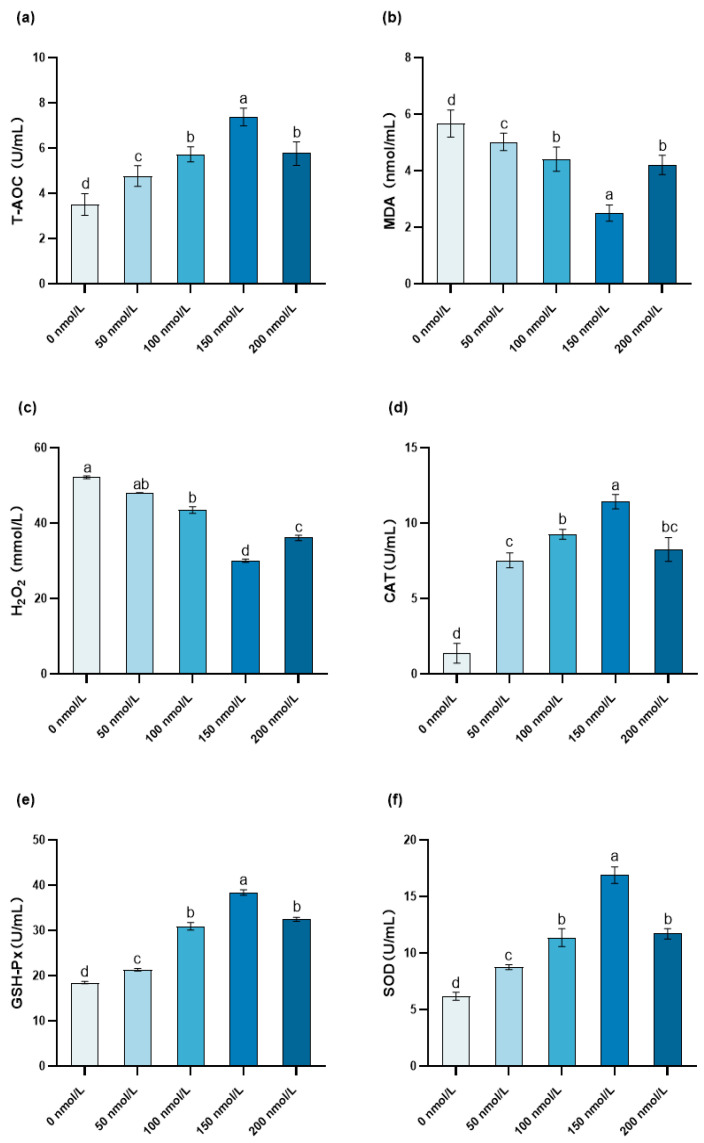
Effect of MitoQ on the antioxidant enzyme activity of boar sperm. (**a**) T-AOC, (**b**) MDA content, (**c**) H_2_O_2_ content, (**d**) CAT activity, (**e**) GSH-Px activity, (**f**) SOD activity. The different letters indicate *p* < 0.05.

Compared with the MitoQ-treated groups at other concentrations, in the 150 nmol/L MitoQ group, the T-AOC capacity of the boar sperm was notably higher (*p* < 0.05). As compared with the 0 nmol/L MitoQ group, there was a significant reduction in the H_2_O_2_ content of the boar sperm in the 150 nmol/L MitoQ group (*p* < 0.05). The addition of MitoQ at concentrations of 50, 100, 150, and 200 nmol/L significantly reduced the MDA content (*p* < 0.05). Among these groups, the 150 nmol/L MitoQ group had a significantly lower MDA content than the other groups (*p* < 0.05). By comparing the 150 nmol/L MitoQ group and the 0 nmol/L MitoQ group, it emerged that the CAT activity in the boar sperm of the 150 nmol/L MitoQ group demonstrated a significant upward tendency (*p* < 0.05). Moreover, the GSH-Px and SOD activities in the 150 nmol/L MitoQ group were remarkably higher than those in the 0 nmol/L MitoQ group (*p* < 0.05) (Figure 4).

### 3.5. The Effect of MitoQ on Sperm Protein Expression

**Figure 5:**
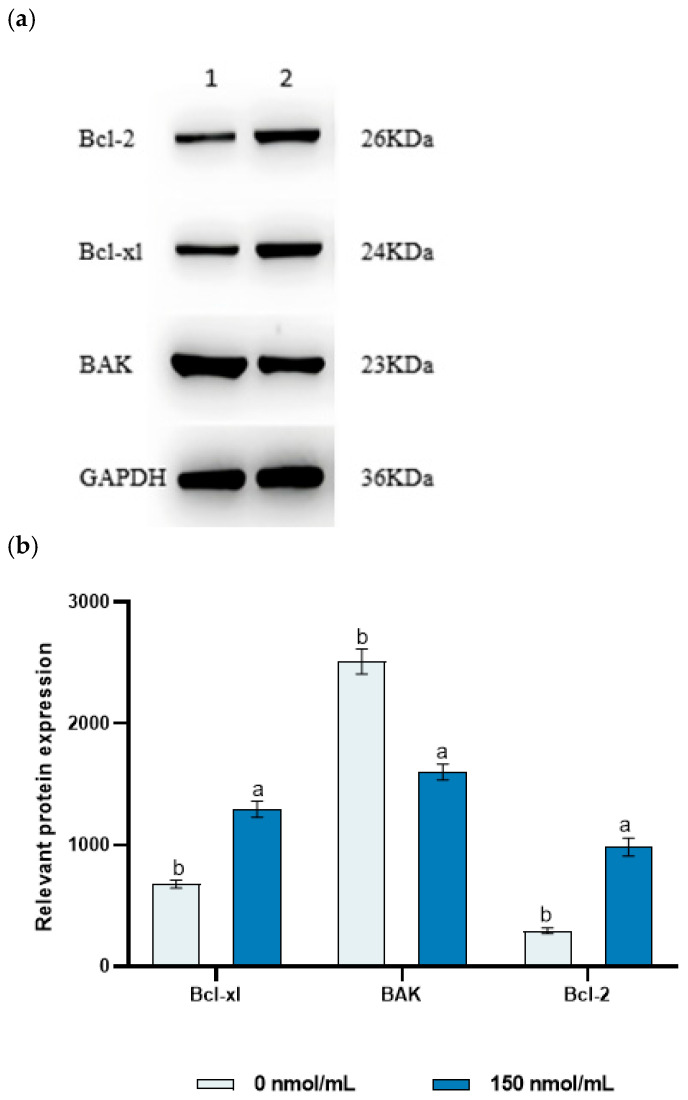
The effects of MitoQ on protein expression after freezing. (**a**) Analysis of Bcl-xl, BAK, and Bcl-2 protein levels using protein blotting. (**b**) Bcl-xl: Antiapoptotic protein, BAK: Bcl-2 related apoptosis protein, Bcl-2: Apoptosis regulatory factor. ^a,b^ Mean values with different superscripts within columns are significantly different at *p* < 0.05

Based on our previous experimental results, we selected sperm samples treated with the optimal concentration of 150 nmol/L MitoQ and sperm samples treated with 0 nmol/L MitoQ to detect sperm-related apoptotic proteins. Compared with the 0 nmol/L MitoQ group, in the 150 nmol/L MitoQ-treated group, the expression level of the antiapoptotic protein Bcl-xl and Bcl-2 in the sperm was significantly increased, and the expression levels of the apoptotic protein BAK were significantly decreased (*p* < 0.05) (Figure 5).

### 3.6. The Effect of MitoQ on the Sperm Adhesion Index

Using the outcomes of prior experiments, the sperm samples treated with the optimal concentration of 150 nmol/L MitoQ were selected, along with those treated with 0 nmol/L MitoQ, for assessing the sperm adhesion index. Compared with the 0 nmol/L MitoQ group, in the 150 nmol/L MitoQ-treated group, the sperm adhesion index was significantly increased (*p* < 0.05) (Table 2).

## 4. Discussion

During boar sperm cryopreservation, sperm produce a large amount of ROS, which weakens sperm motility and causes DNA damage. This also triggers intense oxidative stress reactions, hindering fertilization and leading to sperm cell death [28]. Research has indicated that incorporating proteins, antioxidants, and cryoprotectants into the cryopreservation extender is beneficial for enhancing the quality of boar semen subsequent to freezing and thawing processes, with antioxidants being a current research hotspot. Simultaneously, the suppression and alleviation of oxidative stress are regarded as crucial elements influencing the quality of sperm after thawing [29]. The antioxidant effects of MitoQ have been widely recognized [30]. However, it is unclear whether they have a beneficial role in the cryopreservation of boar sperm. This experiment explored the effects of adding different concentrations of MitoQ to the cryo-diluent on the cryopreservation of semen. The findings indicated that MitoQ had a significant impact in enhancing the cryopreservation quality of boar sperm and augmenting its antioxidant ability, with 150 nmol/L being the optimal concentration.

Recent studies have found that mitochondria are key to maintaining sperm function and vitality [31]. The mitochondria produce ATP through oxidative phosphorylation (OXPHOS) to support cellular activities [32]. Research has shown that ATP produced by mitochondrial OXPHOS can provide energy for goat sperm motility [33]. Being a main source of ROS, mitochondrial ROS (mtROS) is a vital factor contributing to cell damage [34]. ROS are by-products of ATP generation by mitochondria. Excessive accumulation of ROS affects mitochondrial activity, thereby impairing ATP production and reducing sperm motility. Sperm motility stands as one of the most vital metrics for assessing sperm quality. Only sperm displaying high motility are capable of traversing the reproductive tract, arriving at the ampulla of the fallopian tube, and thereby accomplishing fertilization [35]. Moreover, research has found that sperm malformation rates are associated with impaired sperm function and a decrease in pregnancy rates [36]. This study found that adding MitoQ to the extender can effectively improve the quality, malformation rate, and related kinetic parameters of sperm after freezing and thawing, with the most significant effect observed at a concentration of 150 nmol/L. Sperm ASL and VAP are thought to be positively correlated with the fertilization rate [37], and in this study, the ASL and VAP of highly motile sperm were also relatively high. At the same time, previous studies have found that the addition of 200 nM MitoQ to male semen can effectively enhance male sperm motility [38]. Masoudi et al. [39] also demonstrated that MitoQ treatment improved the vitality of ram sperm and concluded that adding MitoQ during in vitro fertilization can enhance the reproductive performance of ewes.

Compared to other species, boar sperm membranes are rich in oxidizable polyunsaturated fatty acids (PUFAs), which stimulate ROS production and increase sperm sensitivity to oxidative stress [40]. Szlasa et al. [41] found high levels of ROS, altering membrane fluidity and permeability, inducing membrane damage, and leading to decreased sperm–oocyte fusion after freezing. Furthermore, the intact acrosome is a crucial prerequisite to ensure the smooth progression of fertilization [42]. Research has found that during oxidative stress, changes in the content of sperm plasma membrane PUFAs hinder the acrosome reaction and reduce the sperm fertilization ability [43]. Mitochondria, as the energy generators in sperm, are related to male germ cell differentiation and gamete function [44]. A decrease in sperm DNA integrity has a negative impact on fertility [45]. Although sperm DNA damage does not hinder the fertilization process of the oocyte, the integrity of sperm DNA is crucial for ensuring normal embryo development [46]. MitoQ, as a mitochondrial-targeted antioxidant, achieves targeted effects by forming a covalent bond with the lipophilic triphenylphosphonium (TPP) cation through its aliphatic carbon chain [47]. Due to the high mitochondrial membrane potential, the cations carried by MitoQ accumulate within the inner mitochondrial membrane. The ubiquinone portion becomes embedded in the lipid bilayer and undergoes redox reactions through the respiratory chain, reducing ubiquinone to ubiquinol [48]. Ubiquinol, as a chain-breaking antioxidant, donates the hydrogen atom from its hydroxyl group to lipid peroxyl radicals, thereby reducing the extent of lipid peroxidation and protecting mitochondria from oxidative stress damage [49]. The research by Rezaei et al. [50] indicates that the addition of 200 nM MitoQ during the cryopreservation of goat semen can reduce the oxidative damage to sperm mitochondria and DNA caused by freezing. In the course of this research, we discovered that the group to which 150 nmol/L MitoQ was added demonstrated a marked elevation in sperm mitochondrial activity and a notable enhancement in acrosome and plasma membrane integrity when contrasted with the group receiving 0 nmol/L MitoQ. A study based on buffaloes showed that the addition of MitoQ improved the body’s antioxidant mechanism, enhanced the mitochondrial membrane potential (δψm), and increased the integrity of the acrosomal plasma membrane [51]. In addition, Moradi et al. [52] found that the addition of MitoQ to male semen can enhance the post-freezing sperm survival rate, ATP content, and acrosome integrity.

T-AOC is used to assess the overall antioxidant levels of semen in relation to sperm vitality [53]. Murawski et al. [54] indicate that a decrease in SOD activity in semen can lead to reduced male fertility or even infertility. MDA is a metabolic product of PUFAs and is a hallmark product of lipid peroxidation [55]. MitoQ is believed to reduce the generation of ROS by activating the Nrf2/ARE signaling pathway, thereby lowering the oxidative stress levels in mitochondria, indicating a potential mechanism by which MitoQ exerts its antioxidant function in sperm [56]. SOD, CAT, and GSH-Px are important enzymes in the sperm antioxidant system, playing a crucial role in the antioxidant process of sperm. In this study, the MitoQ concentration was positively correlated with the GSH-Px, CAT, and SOD activities and negatively correlated with the H_2_O_2_ and MDA content. This is consistent with the findings of Sun et al. [57], in which the addition of 150 nmol/L MitoQ significantly increased the activities of SOD and GSH-Px in rooster sperm after freezing and thawing, improving the redox balance. Sharma et al. [30] found that the addition of MitoQ during in vitro maturation reduces ROS production, improving the maturation of buffalo oocytes and the developmental capacity of cloned embryos. MitoQ alleviates oxidative stress and apoptosis by activating GPX1 and SOD2.

Bcl-xl, BAK, and Bcl-2 are key proteins in the regulatory system of apoptosis, playing a significant role in maintaining the dynamic balance of sperm cell survival and death [58]. Research has shown that oxidative stress can significantly alter the expression and activity of apoptosis-related proteins [59]. When the intracellular ROS levels are excessively high, the pro-apoptotic protein BAK is activated and oligomerizes, disrupting the stability of the mitochondrial membrane and ultimately triggering cell apoptosis [60]. Our research findings indicate that MitoQ may prevent the occurrence of apoptosis by lowering ROS levels and inhibiting or activating the expression of BAK protein. This result is consistent with the findings of Seervi et al. [61], who discovered that N-acetyl-L-cysteine (NAC) can effectively inhibit the activation of BAK and reduce apoptosis by lowering ROS levels during their study on the mechanism of cell death mediated by Thiozolydine (TDZ) in cervical cancer cells. Bcl-xl and Bcl-2 act as antiapoptotic proteins by binding to BAK and inhibiting its activation, preventing changes in the permeability of the outer mitochondrial membrane, thereby averting the release of pro-apoptotic factors such as cytochrome c and inhibiting the transmission of downstream apoptotic signals; Bcl-2 and Bcl-xl inhibit the pro-apoptotic function of BAK through their interaction, thereby enhancing cell survival [62]. The research findings indicate that the addition of MitoQ significantly upregulated the protein expression of Bcl-xl and Bcl-2, enhancing the antiapoptotic capacity of sperm cells, suggesting that MitoQ may promote the survival of sperm cells by regulating the expression of these antiapoptotic proteins. In addition, through sperm penetration experiments, it was observed that the sperm adhesion index using 150 nmol/L MitoQ was higher than that of the control group. This is similar to the findings of Wang et al. [63], in which MitoQ reduced the generation of ROS by downregulating UCP2, thereby promoting human spermatogenesis.

In our research, MitoQ demonstrated good antioxidant capacity and the potential to maintain the quality of frozen sperm. However, our study still has some limitations. The study did not validate the effect of adding MitoQ to frozen sperm on the pregnancy rate of sows in vivo.

## 5. Conclusions

In summary, incorporating MitoQ into the cryopreservation extender is effective in improving the sperm quality and malformation rate, and enhancing sperm kinetic parameters. Furthermore, MitoQ can effectively improve sperm acrosome integrity and mitochondrial activity. With the addition of MitoQ, the antioxidant capacity of sperm is enhanced. The significant increase in the adhesion index further indicates the potential application of MitoQ in real-world production.

## Figures and Tables

**Table 1:** Effect of MitoQ on boar sperm quality and kinetic parameters.

Parameters	0	50	MiotQ (nmol/mL) 100	150	200
Viability (%)	49.75 ± 0.06 ^d^	65.93 ± 0.05 ^c^	73.86 ± 0.13 ^b^	80.83 ± 0.32 ^a^	75.70 ± 0.50 ^ab^
Motility (%)	48.40 ± 0.50 ^c^	53.78 ± 1.55 ^c^	63.30 ± 0.37 ^b^	75.60 ± 0.24 ^a^	65.29 ± 0.20 ^b^
Malformation rate (%)	27.23 ± 0.55 ^d^	20.98 ± 0.05 ^c^	16.70 ± 0.15 ^bc^	11.51 ± 0.16 ^a^	15.07 ± 0.40 ^cd^
VAP (μm/s)	26.29 ± 0.33 ^d^	35.87 ± 0.67 ^c^	40.59 ± 0.13 ^b^	49.98 ± 0.74 ^a^	40.62 ± 1.15 ^b^
VCL (μm/s)	31.89 ± 2.04 ^d^	42.52 ± 1.48 ^d^	46.45 ± 1.17 ^c^	51.73 ± 2.04 ^a^	43.60 ± 0.93 ^cb^
VSL (μm/s)	29.66 ± 1.28 ^c^	39.64 ± 0.33 ^b^	42.72 ± 0.40 ^b^	50.96 ± 1.43 ^a^	42.45 ± 1.49 ^b^
BCF (Hz)	6.24 ± 0.16 ^c^	8.16 ± 0.50 ^bc^	8.67 ± 0.88 ^b^	12.68 ± 1.17 ^a^	8.67 ± 0.52 ^bc^

^a–d^ Mean values with different superscripts within columns are significantly different at *p* < 0.05. Motility: total motility.

**Table 2:** Effect of MitoQ on boar sperm adhesion index.

Group	Number of Oocytes (*n* = 3)	Adhesion Index
0 nmol/mL MiotQ	54	2.67 ± 0.58 ^b^
150 nmol/mL MiotQ	58	5.17 ± 1.26 ^a^

^a,b^ Mean values with different superscripts within columns are significantly different at *p* < 0.05.

## Data Availability

The data in this study can be obtained from the corresponding author upon request.
